# 485. Pediatrics Institutional COVID-19 Review

**DOI:** 10.1093/ofid/ofab466.684

**Published:** 2021-12-04

**Authors:** Prakash Karna, Lauren Farrand, Uzma Hasan

**Affiliations:** 1 Newark Beth Israel Medical Center, Woodbridge Township, New Jersey; 2 Saint Barnabas Medical Center, Green Pond, New Jersey

## Abstract

**Background:**

Coronavirus disease (COVID-19) caused by SARS-COV2 represents global public health concern, with varied severity of illness in different ages and racial groups. This study aims to describe clinical presentation and outcomes in children aged 0-21 years in a community hospital setting in New Jersey.

**Methods:**

This is a retrospective medical record review of pediatric patients (0-21 years) admitted to Saint Barnabas Medical Center between March 2020- December 2020 with confirmed diagnosis of COVID-19 infection. Diagnosis of COVID-19 infection is based on ICD-10 diagnosis code. Data was extracted from electronic medical records, including demographics, pre-existing conditions, presenting symptoms, treatments used and outcomes.

**Results:**

We identified 48 cases of pediatric COVID-19 patients at Saint Barnabas Medical Center during period of 03/20-12/20. Review of demographic data showed 29 patients (60%) were female, and 19 (40%) were male. Race distribution was 38% black, 17% white, 4 % Asian Indian, and 41% others/unknown. Age distribution was as follows: 40% >15 yrs, 15% 11-15 yrs, 15% 0-1 yrs, 13% 6-10 yrs, 13% 1-5 yrs, and 6% newborn. Fever (65%) was the most frequent symptom identified, followed by cough (31%), nausea/vomiting (29%), abdominal pain (19%), shortness of breath (17%), rash (15%), diarrhea (10%), headache (10%), myalgia/body-aches (8%), chest pain (6%), red eyes (6%), and loss of taste/smell (2%). Of 48 patients, 10 (21%) had positive chest X-ray findings of lung infiltrates or opacities, 4 (8%) had abnormal echocardiogram findings, and 1 (2%) had abnormal CT chest. 21 of 48 patients had underlying comorbid conditions, with Diabetes and Asthma being the most common. No deaths were reported. 8 of 48 COVID-19 patients were diagnosed with MIS-C. Of these MIS-C patients, 5 (63%) were male and 3 (38%) were female. 6 of 8 affected patients were black (75%). 50% of MIS-C patients were between 6-10 years. 3 of 8 patients (38%) had abnormal echocardiogram findings.

**Conclusion:**

This review supports clinical findings from other studies and also suggests certain racial ethnicities may be disproportionately impacted, which warrants further exploration to determine genetics vs environmental factors that lead to increased predisposition to severe illness.

**Disclosures:**

**All Authors**: No reported disclosures

